# Overexpression of EphB2 in hippocampus rescues impaired NMDA receptors trafficking and cognitive dysfunction in Alzheimer model

**DOI:** 10.1038/cddis.2017.140

**Published:** 2017-03-30

**Authors:** Rui Hu, Pan Wei, Lu Jin, Teng Zheng, Wen-Yu Chen, Xiao-Ya Liu, Xiao-Dong Shi, Jing-Ru Hao, Nan Sun, Can Gao

**Affiliations:** 1 Jiangsu Province Key Laboratory of Anesthesiology, Xuzhou Medical University, Xuzhou, Jiangsu 221004, China; 2 Jiangsu Province Key Laboratory of Anesthesia and Analgesia Application, Xuzhou Medical University, Xuzhou, Jiangsu 221004, China; 3 Department of Anesthesiology, Xuzhou TCM Hospital, Xuzhou, Jiangsu 221009, China

## Abstract

Alzheimer's disease (AD) is a progressive neurodegenerative disease, which affects more and more people. But there is still no effective treatment for preventing or reversing the progression of the disease. Soluble amyloid-beta (A*β*) oligomers, also known as A*β*-derived diffusible ligands (ADDLs) play an important role in AD. Synaptic activity and cognition critically depend on the function of glutamate receptors. Targeting *N*-methyl-D-aspartic acid (NMDA) receptors trafficking and its regulation is a new strategy for AD early treatment. EphB2 is a key regulator of synaptic localization of NMDA receptors. A*β* oligomers could bind to the fibronectin repeats domain of EphB2 and trigger EphB2 degradation in the proteasome. Here we identified that overexpression of EphB2 with lentiviral vectors in dorsal hippocampus improved impaired memory deficits and anxiety or depression-like behaviors in APPswe/PS1-dE9 (APP/PS1) transgenic mice. Phosphorylation and surface expression of GluN2B-containing NMDA receptors were also improved. Overexpression of EphB2 also rescued the ADDLs-induced depletion of the expression of EphB2 and GluN2B-containing NMDA receptors trafficking in cultured hippocampal neurons. These results suggest that improving the decreased expression of EphB2 and subsequent GluN2B-containing NMDA receptors trafficking in hippocampus may be a promising strategy for AD treatment.

Alzheimer's disease (AD) is a progressive neurodegenerative disease and represents significant and increasing clinical challenge, leading to permanent loss of memory and other cognitive functions.^1, 2^ The amyloid-beta (A*β*), especially soluble A*β*-derived diffusible ligands (ADDLs), seem to be more intimately correlated with cellular and cognitive dysfunction rather than fibril and plagues.^3, 4, 5^ However, the precise mechanisms of A*β*-dependent neural dysfunction and degeneration are still largely unknown and under intense investigation.^1^

Synaptic activity and cognition critically depend on the function of glutamate receptors, which appear to be affected by A*β* oligomers. *N*-methyl-D-aspartic acid (NMDA) receptors have been of particular interest, and play an important role in the cognitive dysfunction of AD.^6, 7^ The function of NMDA receptors is directly dependent on their location and the composition of subunits at synaptic sites.^8^ Soluble A*β* oligomers can alter NMDA receptors equilibrium and activity at synaptic sites.^9, 10, 11, 12^ The protein levels and the phosphorylation status of the NMDA receptor subunits GluN1, GluN2A and GluN2B are shown to correlate with cognitive performance.^13^ Surface expression of GluN2B-containing NMDA receptors and the levels of GluN1 and GluN2B subunits in cortical neurons were found reduced by A*β*1–42.^14, 15, 16^ Tryosine 1472 site (Y1472) was found to be the main phosphorylation site of GluN2B. Src family kinases-mediated tyrosine phosphorylation of NMDA receptor subunits may stabilize NMDA receptors on the cell surface and thereby increase the response to NMDA receptors.^17^ Among these kinases, EphB2 which is co-localized with NMDA receptors both *in vitro* and *in vivo*^18^ is a key regulator of synaptic localization of NMDA receptors^19, 20^ and is found to interact with A*β* oligomers directly.^21, 22, 23^ Human amyloid precursor protein (hAPP) transgenic mice with high brain levels of A*β* oligomers have hippocampal depletions of EphB2.^22^ Increasing EphB2 expression in the dentate gyrus of APP transgenic mice with lentiviral constructs of wild-type (WT) EphB2 reverses the deficits in NMDA receptor-dependent long-term potentiation (LTP) and memory impairment.^21^ Thus, the depletion of EphB2 in the brain should be an important factor for the AD. However, the direct cytology and ethology evidence for overexpression of EphB2 in the dorsal region of hippocampus in AD or animal model and relative mechanisms are still missing.

Thus, a potential therapy targeting the ADDLs-EphB2-NMDA receptors and the subsequent biological cascades could be done by overexpression of EphB2. In the present study, we used a lentiviral vector expressing EphB2-GFP or EphB2-Flag in cultured hippocampal neurons and dorsal hippocampus in APP/PS1 transgenic mice. We found that overexpression of EphB2 not only rescued the impaired GluN2B-containing NMDA receptors trafficking induced by ADDLs in cultured hippocampal neurons, but also improved the impaired cognitive functions and GluN2B-containing NMDA receptors trafficking in APP/PS1 transgenic mice. Our data reveal that improving the decreased expression of EphB2 in hippocampus may be a promising strategy for AD treatment.

## Results

### Overexpression of EphB2 improves GluN2B-containing NMDA receptors trafficking in cultured hippocampal neurons

Our recent work showed that ADDLs reduced the expression of EphB2 and impaired GluN2B-containing NMDA receptors trafficking in cultured hippocampal neurons.^23^ In order to investigate the effect of overexpression of EphB2 on the NMDA receptors trafficking, we first confirmed this experiment and got the similar data (see Supplementary Figure 1). These results demonstrated that the ADDLs decreased the total and surface expression of EphB2, as well as the surface expression of GluN2B-containing NMDA receptors. Therefore, 6 h of exposure to ADDLs (500 nM) was used in the subsequent experiments.

**Figure 1:**
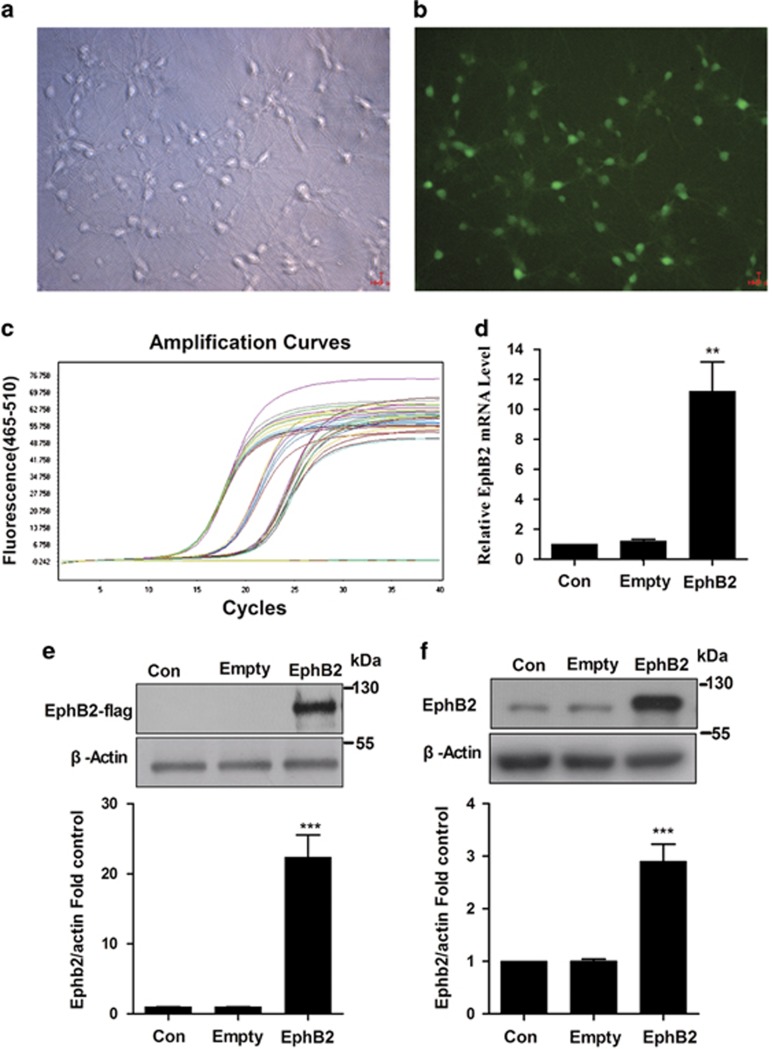
Overexpression of EphB2 with lentivirus upregulates the level of EphB2 in primary hippocampal neurons. (**a** and **b**) Primary hippocampal neurons were well infected with Lenti-GFP. (**c** and **d**) qRT-PCR application and quantification of EphB2-Flag mRNA showed overexpression of EphB2 infected with Lenti-EphB2 (*n*=5 in each group). (**e** and **f**) The expression of exogenous and endogenous EphB2 proteins showed overexpression of EphB2 infected with Lenti-EphB2 with anti-Flag and EphB2, respectively (*n*=6 in each group). **P<0.01, ***P<0.001 *versus* control group. Data are presented as mean±S.E.M.

**Figure 2:**
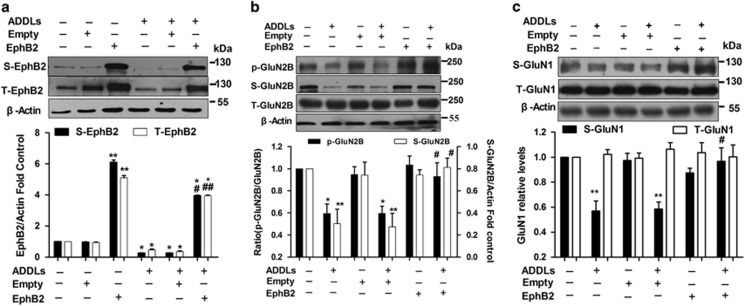
Overexpression of EphB2 rescues decreased expressions of EphB2 and the surface expression of GluN2B-containing NMDA receptors induced by ADDLs in cultured hippocampal neurons. (**a**) Overexpression of EphB2 rescued the decreased total and surface expression of EphB2 induced by ADDLs (*n*=3 in each group). (**b**) Overexpression of EphB2 rescued the decreased surface expression of GluN2B. The reduced pY1472 was improved as well (p-GluN2B: *n*=5 in each group, S-GluN2B: *n*=3 in each group). (**c**) Overexpression of EphB2 rescued the decreased surface expression of GluN1 (*n*=4 in each group). *P<0.05, **P<0.01 *versus* corresponding control group; ^#^*P*<0.05, ^##^*P*<0.01 *versus* corresponding ADDLs group. Data are presented as mean±S.E.M.

To determine whether overexpression of EphB2 could improve the phosphorylation level of GluN2B at Y1472 and subsequent trafficking to the membrane, cultured hippocampal neurons were infected with lentiviral vectors expressing green fluorescent protein (GFP) or flag with EphB2 (Lenti-EphB2, LV) or vacant vector (Lenti-empty, VV). We found that the most efficient MOI value was 10 (Figures 1a and b). The mRNA and protein levels of EphB2 were confirmed by qRT-PCR and western blot. Both the mRNA (mRNA: *F*_(2,18)_=16.294, *P*=0; Figures 2c and d) and protein levels (T-EphB2-Flag: *F*_(2,15)_=45.362, *P*=0; T-EphB2-GFP: *F*_(2,24)_=33.494, *P*=0; Figures 1e and f) of EphB2 were increased upon Lenti-EphB2 treatment, while Lenti-empty had no effect (Figures 1c–f).

Next, we investigated the effect of overexpression of EphB2 on the surface and total expression of EphB2, both of which were significantly reduced by ADDLs. Lenti-empty itself had no significant effect compared with control group. After treatment with Lenti-EphB2, both the surface and total expression of EphB2 were rescued (S-EphB2: *F*_(5,12)_=1.572, *P*=0; T-EphB2: *F*_(5,12)_=882.54, *P*=0; Figure 2a). Then, we examined the effects of overexpression of EphB2 on NMDA receptor subunits expression. The decreased surface expressions of both GluN2B and GluN1 induced by ADDLs were remarkably rescued upon Lenti-EphB2 treatment (S-GluN2B: *F*_(5,12)_=7.006, *P*=0.003 ; S-GluN1: *F*_(5,12)_=9.120, *P*=0 (Figures 2b and c). Similarly, the decreased pY1472 of GluN2B was also rescued by Lenti-EphB2 as well (*F*_(5,22)_=6.957, *P*=0; Figure 2b).

### Overexpression of EphB2 in hippocampus ameliorates impaired learning and memory in APP/PS1 mice

**Figure 3:**
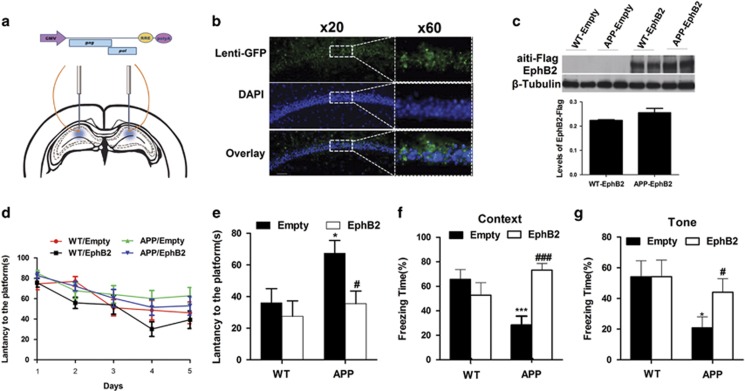
Overexpression of EphB2 in hippocampus ameliorates learning and memory deficits in APP/PS1 transgenic mice. (**a** and **b**) The lentivirus was injected into dorsal hippocampus and the neurons were well infected with Lenti-GFP. (**c**) Quantification of EphB2-Flag expression showed no difference between APP/PS1 transgenic mice and WT mice (*n*=9–10). (**d** and **e**) APP/PS1 mice injected with Lenti-EphB2 spent less time on reaching the platform than that of Lenti-empty after training (*n*=10 in each group). The latency to first reach the platform was recorded 24 h after last training session. Lenti-empty-treated APP/PS1 transgenic mice spent more time on reaching the platform compared to corresponding WT mice. While Lenti-EphB2-treated APP/PS1 transgenic mice performed less time. (*n*=10 in each group). (**f**) Overexpression of EphB2 significantly improved the impaired context-dependent fear memory in APP/PS1 transgenic mice (*n*=10 in each group). (**g**) Overexpression of EphB2 significantly improved the impaired tone-dependent fear memory in APP/PS1 transgenic mice (*n*=10 in each group). *P<0.05, ***P<0.001 *versus* Lenti-empty-treated WT group; ^#^*P*<0.05, ^###^*P*<0.001 *versus* Lenti-empty-treated APP group. Data are presented as mean±S.E.M.

To determine whether overexpression of EphB2 in dorsal hippocampus rescued impaired learning and memory in APP/PS1 mice, Lenti-EphB2-Flag, Lenti-empty-Flag or Lenti-GFP (0.25 *μ*l per side) was injected bilaterally into the dorsal hippocampus of 6-month-old APP/PS1 transgenic mice (APP) and WT mice for 1 month (Figure 3a). One month later, the GFP was well expressed in dorsal hippocampus (Figure 3b). First we measured the distribution and expression of EphB2-Flag in WT and APP/PS1 transgenic mice injected with Lenti-empty or Lenti-EphB2-Flag. There was no significant difference between WT and APP/PS1 transgenic mice (T-EphB2-Flag: *F*_(2,4)_=0.1598, *P*=0.482; Figure 3c).Then the mice were trained and tested in the Morris water maze (MWM) test and fear conditioning. All animals were trained in the MWM test for 5 days. After the second trial day, APP/PS1 transgenic mice injected with Lenti-empty spent much more time on reaching the platform compared to WT mice, while APP/PS1 transgenic mice injected with Lenti-EphB2 were almost indistinguishable from the WT mice in the acquisition task (groups: *F*_3=_3.601, *P*=0.024; days: *F*_4_=22.619, *P*<0.001; Figure 3d). On the test day, the Lenti-EphB2-treated APP/PS1 transgenic mice spent less time on reaching the original platform location than Lenti-empty-treated APP/PS1 transgenic mice (*P*=0.037; Figure 3e), while the Lenti-EphB2-treated WT mice performed similarly to Lenti-empty-treated WT mice.

In the fear-conditioning test, the EphB2-overexpressed group presented a similar protective effect (Figures 3f and g) to MWM. Overexpression of EphB2 rescued both the impaired context (F_(3,37)_=8.917, *P*=0; Figure 3f) and tone (*F*_(3,34)_=5.449, *P*=0.04; Figure 3g)-dependent fear memory in APP/PS1 transgenic mice.

### Overexpression of EphB2 in hippocampus ameliorates anxiety- or depression-like behaviors in APP/PS1 transgenic mice

**Figure 4:**
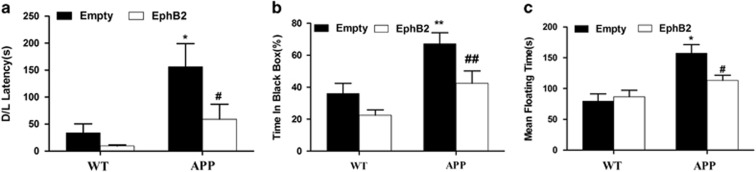
Overexpression of EphB2 in hippocampus ameliorates anxiety- or depression-like behaviors in APP/PS1 transgenic mice. (**a** and **b**) Overexpression of EphB2 ameliorated anxiety-like behavior. APP/PS1 transgenic mice showed an anxiety-like behavior. Lenti-EphB2-treated but not Lenti-empty-treated APP/PS1 transgenic mice spent less time on the latency (**a**) and dark area (**b**) (*n*=10 in each group). (**c**) APP/PS1 transgenic mice showed a depression-like behavior. Lenti-EphB2-treated but not Lenti-empty-treated APP/PS1 transgenic mice spent less time on floating time (*n*=10 in each group). *P<0.05, **P<0.01 *versus* Lenti-empty-treated WT group; ^#^*P*<0.05, ^##^*P*<0.01 *versus* Lenti-empty-treated APP group. Data are presented as mean±S.E.M.

Anxiety disturbances have been reported in some of the AD mouse models.^24, 25, 26, 27, 28, 29^ We next examined whether the overexpression of EphB2 could affect anxiety- or depression-like behaviors in APP/PS1 transgenic mice. These behaviors were evaluated by dark/light emergence and forced swim test, respectively. APP/PS1 transgenic mice showed an anxiety-like behavior (latency: *F*_(3,34)_=5.310, *P*=0.004; time in black area: *F*_(3,34)_=9.021, *P*=0; Figures 4a and b). Lenti-EphB2-treated but not Lenti-empty-treated APP/PS1 transgenic mice spent less time on the latency (*P*=0.048) and dark area (*P*=0.009). There was a trend toward the decrease of the latency (*P*=0.665) and time exploring in dark area (*P*=0.194) in WT mice when treated with Lenti-EphB2, but there was no significant difference. Forced swim test showed that APP/PS1 transgenic mice spent more time on floating than that of WT mice (*F*_(3,36)_=7.956, *P*=0.001; Figure 4c). Lenti-EphB2-treated but not Lenti-empty-treated APP/PS1 transgenic mice spent similar time on floating to WT mice (*P*=0.04). These results showed that overexpression of EphB2 could ameliorate anxiety- or depression-like behaviors in APP/PS1 transgenic mice.

### Overexpression of EphB2 in hippocampus rescues the decreased total and surface expression of EphB2, as well as the GluN2B-containing NMDA receptors trafficking in APP/PS1 transgenic mice

**Figure 5:**
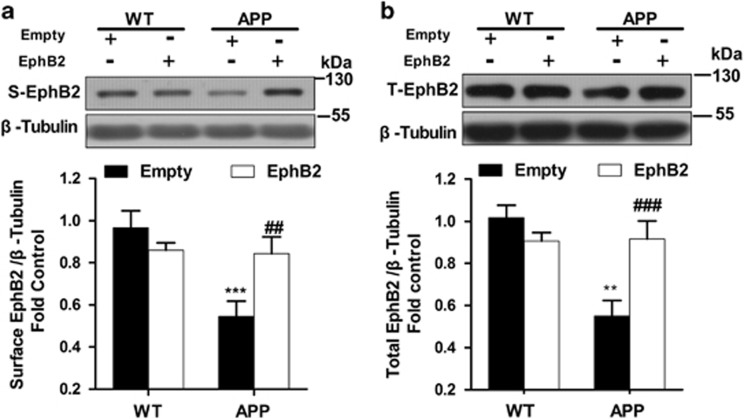
Overexpression of EphB2 in hippocampus rescues the expression of EphB2 in APP/PS1 transgenic mice. (**a**) Overexpression of EphB2 significantly rescued the reduced total expression of EphB2 in APP/PS1 transgenic mice (*n*=4 in each group). (**b**) Overexpression of EphB2 significantly improved the surface expression of EphB2 in APP/PS1 transgenic mice (*n*=4 in each group). **P<0.01, ***P<0.001 *versus* the Lenti-empty-treated WT group; ^##^*P*<0.01, ^###^*P*<0.001 *versus* the Lenti-empty-treated APP group. Data are presented as mean±S.E.M.

To further confirm the protective mechanisms for overexpression of EphB2 in APP/PS1 transgenic mice, we next examined the effect of overexpression of EphB2 on NMDA receptors trafficking. First, we measured the expression of the total and surface expression of EphB2 in the hippocampus of APP/PS1 transgenic mice and WT mice. Both the surface (*P*=0.001; Figure 5a) and the total (*P*=0.008; Figure 5b) expressions of EphB2 were decreased in APP/PS1 transgenic mice treated with empty vector, but were significantly rescued upon EphB2 overexpression (S-EphB2: *F*_(3,16)_=6.742, *P*=0.004; T-EphB2: *F*_(3,16)_=9.338, *P*=0.001; Figures 5a and b).

**Figure 6:**
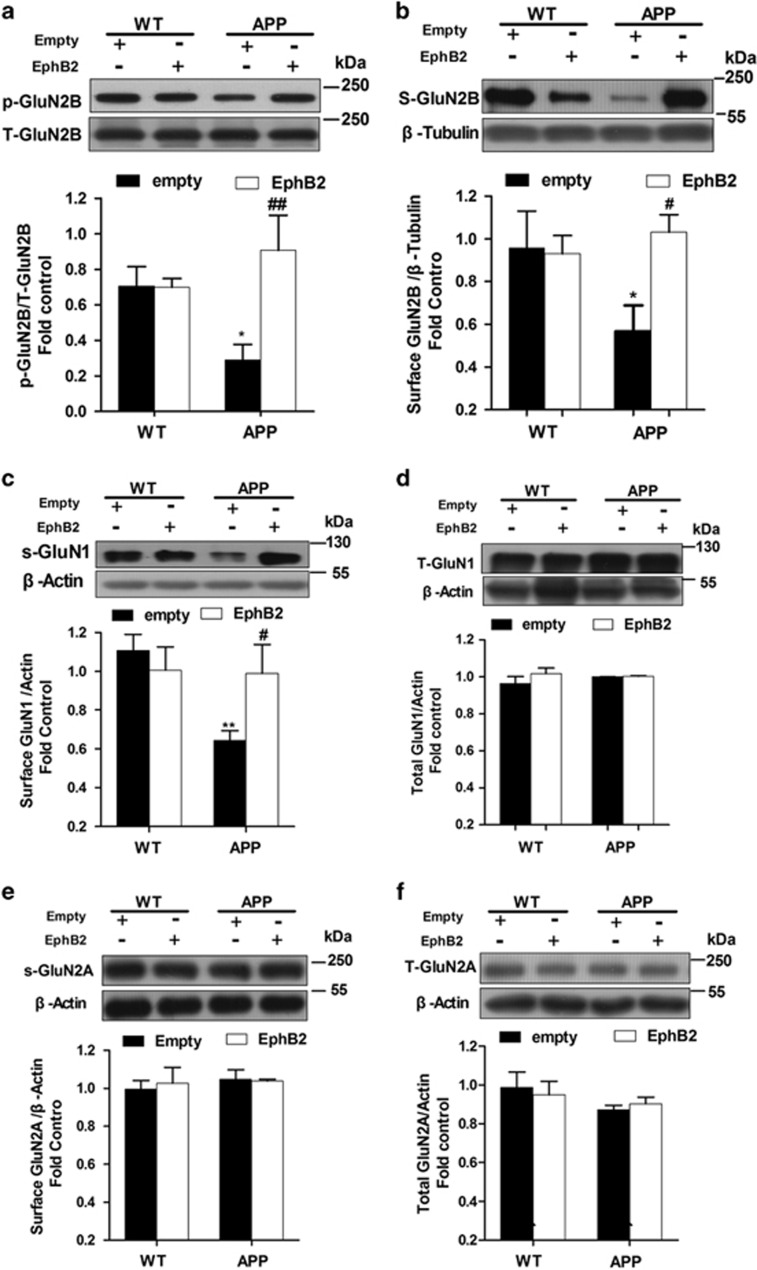
Overexpression of EphB2 in hippocampus rescues the GluN2B-containing NMDA receptors trafficking in APP/PS1 transgenic mice. (**a**) Overexpression of EphB2 significantly rescued the reduced phosphorylated level of GluN2B at pY1472 in APP/PS1 transgenic mice (*n*=4 in each group). (**b**) Overexpression of EphB2 significantly rescued the reduced surface expression of GluN2B in APP/PS1 transgenic mice (*n*=4 in each group). (**c**) Overexpression of EphB2 significantly rescued the reduced surface expression of GluN1 in APP/PS1 transgenic mice (*n*=4 in each group). (**d**) Overexpression of EphB2 had no effect on the total expression of GluN1 in APP/PS1 transgenic mice (*n*=4 in each group). (**e** and **f**) Overexpression of EphB2 had no effect on both the total (**e**) and surface (**f**) expressions of GluN2A in APP/PS1 transgenic mice (*n*=4 in each group). *P<0.05, **P<0.01 *versus* the Lenti-empty-treated WT group; ^#^*P*<0.05, ^##^*P*<0.01 *versus* the Lenti-empty-treated APP group. Data are presented as mean±S.E.M.

Similarly, overexpression of EphB2 remarkably rescued the decreased pY1472 of GluN2B (*F*_(3,15)_=5.317, *P*=0.012; Figure 6a) and surface expression of GluN2B in APP/PS1 transgenic mice (*F*_(3,19)_=2.368, *P*=0.048; Figure 6b). As expected, Lenti-EphB2 treated had a similar protective effect on the decreased surface expression of GluN1(S-GluN1: *F*_(3,20)_=3.551, *P*=0.033; Figure 6c) but had no effect on the total expression of GluN1 (T-GluN1: *F*_(3,11)_=0.940, *P*=0.454; Figure 6d). Both surface and total expressions of GluN2A were not changed in APP/PS1 transgenic mice (S-GluN2A: *F*_(3,8)=_0.181, *P*=0.906; T-GluN2A: *F*_(3,20)_=0.776, *P*=0.521; Figures 6e and f) supporting our finding that ADDLs did not affect the surface expression of GluN2A in cultured hippocampal neurons (see Supplementary Figure S1). These results indicate that the exogenous expression of EphB2 can reverse impaired GluN2B-containing NMDA receptors trafficking in AD models.

## Discussion

Overall, we demonstrated that reversing EphB2 expression by lentiviral vector in the dorsal hippocampus rescued cognitive deficits, the phosphorylation and surface expression of GluN2B-containing NMDA receptors in APP/PS1 transgenic mice. We also found that overexpression of EphB2 improved EphB2 expression and the subsequent effect on GluN2B phosphorylation and trafficking induced by ADDLs in cultured hippocampal neurons.

Increasing evidence links dysregulation of NMDA receptors trafficking to AD.^14, 30, 31^ A*β* oligomers disturb the function of GluN2B-containing NMDA receptors and associated signaling molecules and impair both glutamatergic transmission and synaptic plasticity. Surface expression of GluN1 in cortical neurons is found to be reduced by A*β* via the activation of the *α*-7 nicotinic receptor and the tyrosine phosphatase STEP.^14^ Using STEP inhibitors may be a novel anti-AD molecule.^32^ Levels of GluN1 and GluN2B subunits are reduced in the hippocampus in AD.^15, 18^ In addition, hAPP transgenic mice carrying high brain levels of A*β* oligomers have decreased phosphorylation levels of GluN2B in the hippocampus.^33^ GluN2B phosphorylation at Y1472 is important for GluN1/GluN2B trafficking to the cell member, which plays an important role in NMDA receptor-dependent synaptic plasticity.^17, 23^ Our results confirmed that ADDLs decreased GluN2B phosphorylation and the surface expression of GluN2B-containing NMDA receptors. Similar results were obtained in APP/PS1 transgenic mice. EphB2 is one of the kinases, which could mediate tyrosine phosphorylation of GluN2B at Y1472 and stabilize NMDA receptors on the cell surface and thereby increase the response of NMDA receptors.^17^ Increasing EphB2 expression in the dorsal hippocampus of APP transgenic mice with lentiviral vector reversed the deficits in memory impairment and anxiety- or depression-like behaviors by recovering GluN2B-containing NMDA receptors trafficking. Similar results showed that increasing EphB2 expression in a subset of granule cells improved dentate gyrus NMDA receptor-dependent LTP and learning and memory in hAPP mice.^21^ These indicate that EphB2 is involved in the dysfunction of the trafficking of GluN2B-containing NMDA receptors and synaptic plasticity in AD.^1^

Synaptic and extrasynaptic NMDA receptors activation have distinct consequences on synaptic plasticity, gene regulation and neuronal death.^34, 35^ A*β* oligomers downregulate the synaptic NMDA receptors function by promoting NMDA receptors endocytosis due to the decreased GluN2B phosphorylation. It can also activate extrasynaptic NMDA receptors to reduce LTP.^36^ Our results demonstrated that blocking extrasynaptic NMDA receptors by memantine ameliorated ADDLs-induced dysfunction of GluN2B-containing NMDARs trafficking by improving the phosphorylation and surface expression of GluN2B, and prevented downregulation of ERK/CREB signaling as well as memory deficits in APP/PS1 transgenic mice (unpublished data).

EphB2 expression was also found decreased in human AD patients and hAPP transgenic mice.^22^ Mice lacking EphB2 have impaired NMDA receptor-dependent LTP and memory deficits.^37, 38^ In mature neurons, EphB2 can enhance the localization of GluN2B-containing NMDA receptors at synapses.^19^ Increasing EphB2 expression drastically improved the activity of LTP and NMDA receptors in hAPP transgenic mice.^21^ EphB2 may exert its effect via phosphorylation of Y1472 at GluN2B due to its tyrosine kinase activity.^17^ In the present study, we found that overexpression of EphB2 could improve impaired learning and memory in APP/PS1 mice including spatial memory (MWM) and associative learning (fear conditioning). Interestingly, lentiviral vector overexpressed EphB2 injected into the dorsal hippocampus not only rescued impaired context-dependent fear conditioning, which is correlated with both the hippo7campus and the amygdale,^39^ but also reversed tone-dependent fear conditioning. The amygdala is believed to be involved in tone-dependent fear conditioning.^40, 41, 42^

Anxiety disturbances have been reported in some of the AD mouse models.^24, 25, 26, 27, 28, 29^ Usually it starts early at 3–6 months in APP/PS1 models. We have also reported that anxiety-like behavior was found in 9-month-old APP/PS1 mice.^43^ In the present study, we found an anxiety-like behavior in APP/PS1 mice at 6–7 months old. Lentiviral vector overexpressed EphB2 injected into the dorsal hippocampus relieved the anxiety-like behaviors. The EphB2-NMDA receptor interaction and downstream signaling in the amygdala may be involved in anxiety.^44^ It remains to be determined whether the hippocampus-amygdala circuit participates in memory deficits and anxiety behaviors in AD.

The depressive symptoms/behaviors are a very common comorbidity with AD, very little work has been devoted to determine the range of depressive behavioral symptoms in the commonly used mouse models of AD.^45, 46^ In the present study, we found the depression-like behavior in the forced swim assay at 6–7 months old. In our previous work with 9-month-old APP/PS1 mice, there was just a trend toward increased duration of immobility in the forced swim test.^43^ Interestingly, lentiviral vector overexpressed EphB2 injected into the dorsal hippocampus relieved the depression-like behaviors in APP/PS1 mice. Hippocampus is one of the brain regions, which are relevant to major depression.^47, 48, 49, 50^ A very recent study found that (2R,6R) hydroxynor ketamine, a non-competitive, glutamatergic NMDA receptor antagonist metabolite, exerts behavioral, electroencephalographic, electrophysiological and cellular antidepressant-related actions in mice.^51^ These antidepressant actions involve early and sustained activation of AMPA receptors in CA1 region of hippocampal. Whether overexpression of EphB2 in dorsal region of hippocampal could activate AMPA receptor to improve depressant actions in AD animals should be further tested.

Gene therapy has the potential to ‘permanently' correct disease by bringing in a normal gene to correct a mutant gene deficiency, knocking down mRNA of mutant alleles, and inducing cell death in cancer cells using transgenes encoding apoptosis-inducing proteins.^52, 53, 54, 55^ Gene therapy to treat human disease has seen its ups and downs over the past 20 years. Through a dedicated research effort, many of the challenges to success are being overcome and there have been several promising clinical trials showing efficacy. Due to the constraints imposed by the blood–brain barrier, the most common delivery route is direct injection into the target region in the brain, which bypasses this barrier. Current generations of lentiviral vectors have a convincing safety record, offering a number of features that promise both increased potency and safety of therapeutic gene transfer into long-lived and replicating target cells. It has been shown that EphB2 level in the hippocampus of AD patients or APP/PS1 transgenic mice is decreased,^22^ and increasing EphB2 levels in the dentate gyrus reverse the deficits in memory impairments in APP/PS1 transgenic mice.^21^ Here we found that increasing EphB2 expression in the dorsal hippocampus of APP transgenic mice with lentiviral vector also reversed the deficits in memory impairment and improved the anxiety- or depression-like behaviors. This may be a promising genetic therapy in AD.

In summary, our studies suggest that reversing EphB2 expression in dorsal hippocampus could rescue the cognitive dysfunction in AD via improving GluN2B-containing NMDA receptors trafficking. This may be a promising strategy for AD treatment.

## Materials and methods

### Animals

Male APP/PS1 mice and WT mice (nontransgenic littermates of APP/PS1 mice) were obtained from Model Animal Research Center of Nanjing University (Nanjing, China). APP/PS1 mice expressed both a chimeric mouse/human APP (Mo/Hu APP 695swe) and a mutant human PS1 (PS1-dE9). All the mice were housed under standard conditions with two–four animals per cage, and were kept in a room (22±2 °C) maintained on a dark–light cycle of 12 h (0800 AM–8:00 PM) with free access to food and water. All studies were approved by the Animal Care and Use Committee of Xuzhou Medical University in compliance with National Institutes of Health standards.

### Drugs

Synthetic A*β*1-42 peptides (Sangon Biotech, Shanghai, China) were lyophilized in 1,1,1,3,3,3-Hexafluoro-2-propanol (HFIP, Sigma-Aldrich, St. Louis, MO, USA) and prepared for ADDLs as described previously.^23^

### Lentivirus construction and stereotaxic injection

Lentivirus-expressing EphB2 (GV287-EphB2) was constructed by Songlei Company (Shanghai, China). To increase the expression of EphB2, a sequence encoding EphB2-Flag was inserted between the MCS and SV40. WT and APP/PS1 transgenic mice were anesthetized by intraperitoneal injection with Avertin (Isoamyl alcohol, 250 mg/kg, i.p.) Mice were placed in a stereotaxic frame, and lentiviral vectors were stereotactically injected bilaterally into the dorsal region of hippocampus (3*μ*l per site) at the following coordinates: a/p, −1.5, m/l±1.0, d/v, –2.0. Behavioral tests were carried out 4 weeks after lentivirus injection.

### Hippocampal cultures and treatments

The hippocampi from 18–20 embryonic Sprague–Dawley rats were isolated and dissociated with trypsin.^23^ Cells were plated on coverslips coated with poly-D-lysine in 96-well culture plates or 6-well culture plates and maintained in NeuroBasal medium with B27 (Invitrogen, Carlsbad, CA, USA). Half of the medium was replaced with identical medium every 4 days. Cultures were kept at 37 °C in a humidified incubator with 5% CO_2_/95% air, and used after 14 days in *vitro*. Neurons at 5 days in *vitro* were infected with Lenti-EphB2-Flag or Lenti-EphB2-GFP in combination with lentiviral constructs. After stimulation with 500 nM ADDLs in the presence or absence of lentivirus-overexpressing EphB2 or vacant lentivirus, cultures were collected at indicated time points in cold homogenization buffer. The samples were stored at −80 °C if not used immediately and sonicated for subsequent determination of protein concentration and western blot analysis. Membrane fractions were prepared by using the ProteoExtract kit (Calbiochem, Darmstadt, Germany) for subcellular proteome extraction according to the instructions.

### qRT-PCR

For quantitative fluorogenic RT-PCR (qRT-PCR), total RNA was isolated with RNeasy Mini kits with an on-column RNase-free DNase I treatment (Takara). Total RNA was reverse transcribed with random hexamers and oligo (dT) primers. Diluted reactions were analyzed with SYBR green PCR reagents (Takara, Dalian, China). Endogenous mouse EphB2 and exogenous EphB2-Flag mRNA empty were normalized to GAPDH. The following primers were used: mouse EphB2 forward, 5′-GTGTGGAGCTATGGCATCGT-3′ reverse, 5′-TGGGCGGAGGTAGTCTGTAG-3′ Flag; forward, 5′-ATTCTGCTGGCTGCTGCT-3′ reverse, 5′-CGTTGCTGTCGTAGAGTCC-3′.

### Immunoblotting

After determination of the protein concentration, protein samples were separated by SDS-PAGE and subsequently transferred to PVDF membranes (Bio-Rad, Hercules, CA, USA). Membranes were blocked by 5% non-fat milk in TBST, then incubated at 4 °C overnight with the following primary antibodies: EphB2 (1:1000; R&D Systems, Minneapolis, MN, USA), GluN1 (1:1000; Invitrogen), GluN2A (1:800; Santa Cruz, Dallas, TX, USA), GluN2B (1:1000; Santa Cruz), P-GluN2B (Y1472) (1:1000; Millipore, Bedford, MA, USA), P-EphB2 (Y594) (1:1000; Abcam, Cambridge, MA, USA), *β*-actin (1:2000; Santa Cruz), GAPDH (1:1000) and His-tag (1:1000; Zhongshan Goldenbridge Biotechnology, Beijing, China). After rinses with TBST, membranes were incubated with horseradish peroxidase-conjugated secondary antibodies (1:1000; Beyotime Institute of Biotechnology, Haimen, China). Protein bands were visualized with an ECL detection system (Beyotime Institute of Biotechnology) and quantified densitometrically with ImageJ software (NIH).

### MWM

The water maze test took place in a round tank (1.2 m in diameter) filled with white opaque water. The day before the task, mice were habituated to the environment. During the training phase, mice were allowed to swim with the platform for 90 s or until they reached the platform, a process monitored by Anymaze software (Stoelting). Animals that did not reach the platform after 90 s were gently guided toward it. All animals were allowed to remain on the platform for 30 s. The training phase lasted for 5 days, with four trials each day. For testing, the latency period for mice to find the platform was recorded 24 h after the last training session from a starting position different from the last starting position used during the training phase.^23^

### Fear conditioning

Contextual and tone-dependent fear conditioning was performed in an automated system (Med Associates, Inc., St. Albans, VT, USA) and consisted of a single exposure to a context (3 min) followed by a 30 s tone (10 kHz; 75 dB SPL) and a foot shock (2 s; 0.7 mA; constant current) as described previously.^56^ Context-dependent freezing was measured 24 h later every 10th second over 180 s by two observers unaware of the experimental conditions and expressed as a percentage of the total number of observations. Freezing to the tone was similarly scored every fifth second in a no-empty context during a 30 s exposure.

### Anxiety

Anxiety-like behavior was evaluated using the dark/light emergence task.^56^ A 10 × 10 cm shelter was placed in the middle of the 60 × 60 cm arena. Latency to come out from the shelter and time spent in the dark area during a 5 min test was recorded automatically by Anymaze software.

### Forced swim test

Mice were placed in an upright cylinder (20 cm diameter) filled with warm water (26 °C) up to 5 cm below its opening. Mice were observed for 6 min, and the amount of time spent in an immobile posture during the last 5 min was scored.^53^

### Data analysis and statistics

Water maze data were analyzed by ANOVA with repeated measure followed by *post hoc* Student-Newman–Keuls multiple comparisons. Other behavior tests and biochemical data were analyzed with one-way ANOVA followed by the Student-Newman–Keuls, least-significant difference (for equal variances) or Dunnett T3 (unequal variances) were used for multiple comparisons. Differences were considered significant when *P*<0.05. Data are shown as the mean values±S.E.M.
