# Supplementary material for: Overexpression of EphB2 in hippocampus rescues impaired NMDA receptors trafficking and cognitive dysfunction in Alzheimer model
Source: Cell Death Dis. 2017 Mar 30;8(3):e2717–. doi: 10.1038/cddis.2017.140 (PMC5386541; doi:10.1038/cddis.2017.140)
Supplement: Supplementary Material [file cddis2017140x1.docx]

**Supplemental Material**

**Results**

**ADDLs reduce the expression of EphB2 and impair GluN2B-containing NMDA receptors trafficking in cultured hippocampal neurons.** In order to investigate the effect of overexpression of EphB2 on the synaptic plasticity and cognition, neurons were incubated with freshly prepared ADDLs (500 nM) for 0, 1, 3, 6, and 24 h. Then, total and surface proteins were extracted and subjected to immunoblotting analysis (Figure 1). Both the total and the surface expression of EphB2 were decreased after 3 h treatment (F_(5,22)_ = 11.536, *P* = 0), with the protein levels reduced to a minimum at 6 h (Figure 1a). As for NMDA receptors, surface expression of GluN2B (Figure 1b) and GluN1 (Figure 1c) showed similar changes to EphB2 after the treatment of ADDLs (S-GluN2B: F_(5,36)_ = 11.520, *P* = 0.001; S-GluN1: F_(5,17)_ = 26.392, *P* = 0.001), but the total amount of GluN2B and GluN1 was not changed (T-GluN2B: F_(5,36)_ = 0.086, *P* = 0.994; T-GluN1: F_(5,17)_ = 0.099, *P* = 0.991; Figure 1b and c). Given that the surface expression of GluN2B-containing NMDA receptors is correlated with their phosphorylated level at Y1472 of GluN2B, we also measured the phosphorylated (pY1472) level of GluN2B. As expected, ADDLs decreased the pY1472 of GluN2B as well (F_(5,23)_ = 3.890, *P* = 0.011; Figure 1b). In contrast, both the total and surface expression of GluN2A was not affected by ADDLs (T-GluN2A: F_(5,12)_ = 0.086, *P* = 0.993; S-GluN2A : F_(5,12)_ = 1.725, *P* = 0.203; Figure 1d). These results demonstrated that the ADDLs decreased the total and surface expression of EphB2, as well as the surface expression of GluN2B-containing NMDA receptors. Therefore, 6 h of exposure to ADDLs was used in the subsequent experiments.

**Legends**

**Figure S1** ADDLs reduce the expression of EphB2 and impair the GluN2B-containing NMDA receptors trafficking in cultured hippocampal neurons. **(a)** The effect of ADDLs on the expression of EphB2. Both the total and surface expression of EphB2 were decreased (n = 5~6 in each group). **(b)** The effect of ADDLs on the expression of GluN2B. The surface expression of GluN2B and the phosphorylated GluN2B at Tyrosine 1472 (Y1472) were reduced, while the total expression of GluN2B was not changed (n = 7 in each group). **(c)** The effect of ADDLs on the expression of GluN1. The surface expression of GluN1 was reduced, but the total expression of GluN1 was not changed (n = 4 in each group). **(d)** The effect of ADDLs on the expression of GluN2A. Both the total and surface expression of GluN2A were not changed (n = 4 in each group). ^*^*P* < 0.05, ^**^*P* < 0.01, ^***^*P* < 0.001 versus corresponding control group (Con). Data are presented as mean ± SEM.
