# Supplementary figures and images for: Overexpression of EphB2 in hippocampus rescues impaired NMDA receptors trafficking and cognitive dysfunction in Alzheimer model
Source: Cell Death Dis. 2017 Mar 30;8(3):e2717–. doi: 10.1038/cddis.2017.140 (PMC5386541; doi:10.1038/cddis.2017.140)

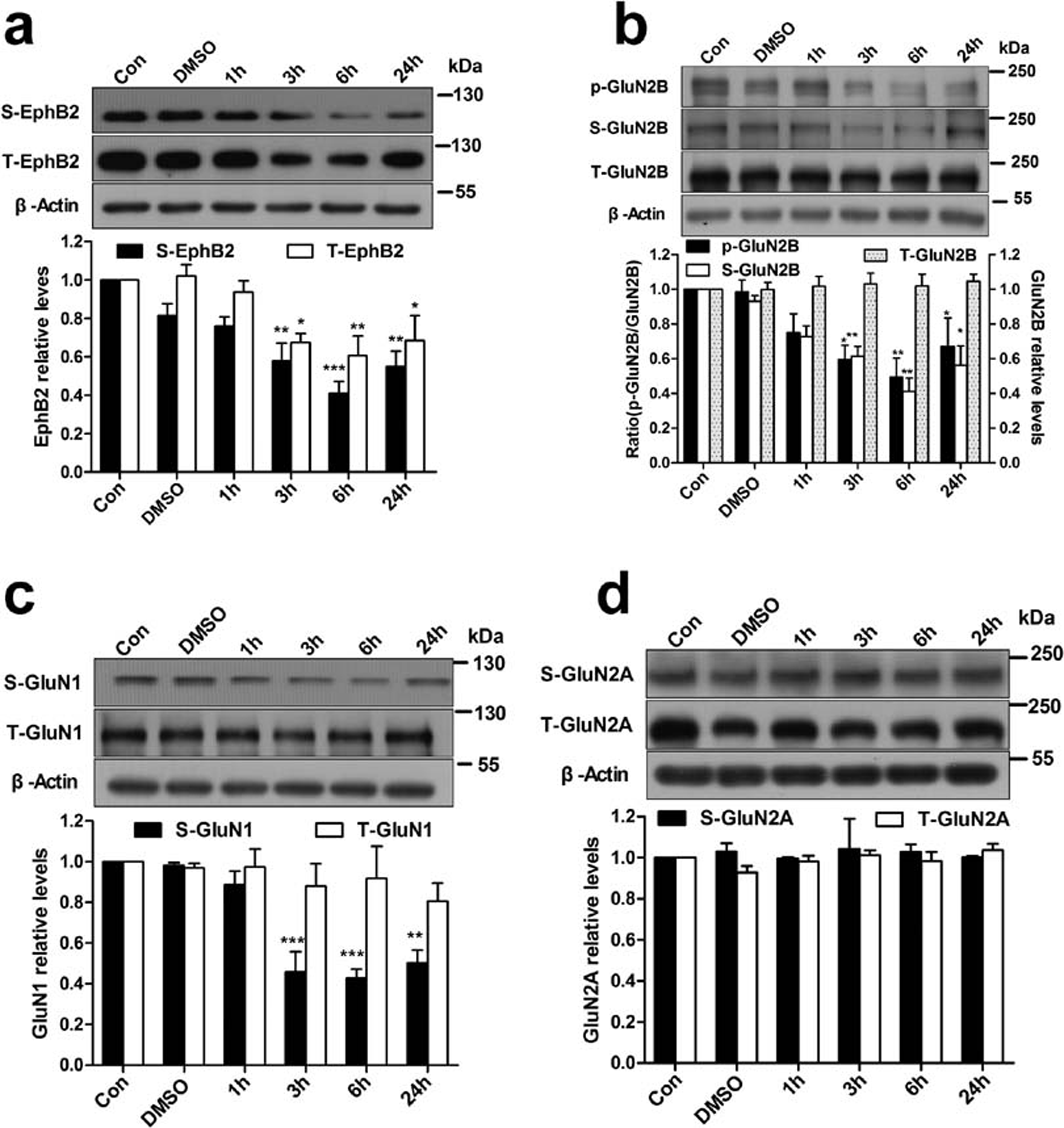

Supplement: Supplementary Figure [file cddis2017140x2.tif]
